# The CCL2-CCR4 Axis Promotes Regulatory T Cell Trafficking to Canine Glioma Tissues

**DOI:** 10.21203/rs.3.rs-4474288/v1

**Published:** 2024-06-17

**Authors:** WK. Panek, RG. Toedebusch, BE. Mclaughlin, PJ Dickinson, JE. Dyke, KD. Woolard, ME. Berens, MS. Lesniak, BK. Sturges, KM. Vernau, C. Li, JM. Miska, CM. Toedebusch

**Affiliations:** University of California, Davis; University of California, Davis; University of California Davis, Flow Cytometry Shared Resource; University of California, Davis; University of California Davis, Flow Cytometry Shared Resource; University of California, Davis; The Translational Genomics Research Institute; Northwestern University; University of California, Davis; University of California, Davis; University of California, Davis; Northwestern University; University of California, Davis

**Keywords:** Dog, Glioblastoma, tumor-infiltrating lymphocyte, CCL2, CCR4

## Abstract

**Purpose:**

Spontaneously occurring glioma in pet dogs is increasingly recognized as a valuable translational model for human glioblastoma. Canine high grade glioma and human glioblastomas share many molecular similarities, including accumulation of immunosuppressive regulatory T cells (Tregs) that inhibit anti-tumor immune responses. Identifying in dog mechanisms responsible for Treg recruitment may afford targeting the cellular population driving immunosuppression, the results providing a rationale for translational clinical studies in human patients. Our group has previously identified C-C motif chemokine 2 (CCL2) as a glioma-derived T-reg chemoattractant acting on chemokine receptor 4 (CCR4) in a murine orthotopic model of glioma. Recently, we demonstrated a robust increase of CCL2 in the brain tissue of canine patients bearing high-grade glioma.

**Methods:**

We performed a series of in vitro experiments using canine Tregs and patient-derived canine glioma cell lines (GSC 1110, GSC 0514, J3T-Bg, G06A) to interrogate the CCL2-CCR4 signaling axis in the canine.

**Results:**

We established a flow cytometry gating strategy for identification and isolation of FOXP3^+^ Tregs in dogs. The canine CD4 + CD25^high^ T-cell population was highly enriched in FOXP3 and CCR4 expression, indicating they are bona fide Tregs. Canine Treg migration was enhanced by CCL2 or by glioma cell line-derived supernatant. Blockade of the CCL2-CCR4 axis significantly reduced migration of canine Tregs. CCL2 mRNA was expressed in all glioma cell lines and expression increased when exposed to Tregs but not to CD4 + helper T-cells.

**Conclusion:**

Our study validates CCL2-CCR4 as a bi-directional Treg-glioma immunosuppressive and tumor-promoting axis in canine high-grade glioma.

## introduction

Despite more than 450 NIH-sponsored phase II and III clinical trials, glioblastoma (GBM) remains a uniformly lethal primary brain tumor of adults [1–4]. Notably, immunotherapeutic approaches that have advanced treatment in several non-CNS cancers have failed to show clinical benefit in GBM [5, 6]. This lack of progress highlights two key challenges that restrain therapeutic advancement across GBM: 1) the microenvironment of adult GBM is markedly immunosuppressed and 2) therapeutic successes in preclinical rodent models poorly translate into increased survival for human patients [7, 8]. The lack of therapeutic translation may rest, in part, on the homogeneous genetic backgrounds and lack of environmental immune influence of rodent models that do not faithfully recapitulate the heterogeneity and complexity of human tumors [8, 9]. There is an urgent need for novel immunotherapeutic approaches to develop and test strategies by which to overcome the immunosuppressed microenvironment of GBM as well as the incorporation of complementary preclinical models which more closely predict therapeutic efficacy in GBM patients [8, 9, 10].

While immunosuppression in GBM is multifactorial, the recruitment of CD4^+^CD25^+^FOXP3^+^ regulatory T cells (Tregs) inhibit anti-tumor immune responses and present a significant therapeutic challenge [9, 10]. We and others have previously shown that glioma derived C-C Motif Chemokine Ligand 2 and 22 (CCL2 and CCL22) is a critical chemoattractant responsible for Treg recruitment into the GBM microenvironment. Moreover, increased intratumoral CCL2 expression correlated with reduced overall survival in human patients [10, 11]. Using murine models, we determined that CCL2 induces Treg recruitment into the glioma microenvironment through the CC chemokine receptor type 4 (CCR4) expressed on Tregs. Importantly, targeting of CCR4 reduced intratumoral Treg abundance and prolonged animal survival [10, 11]. Therefore, the CCL2-CCR4 signaling axis may serve as a therapeutic target for GBM patients.

Canine high-grade gliomas (HGGs) which develop *de novo* in an outbred, immunocompetent host, are increasingly pursued as a therapeutic model for human GBM [12–17]. Canine HGGs, as defined by the Comparative Brain Tumor Consortium of the National Cancer Institute, encompass grade III and IV canine astrocytomas and oligodendrogliomas [12]. With a comparable disease incidence, naturally occurring canine HGGs share clinical, imaging, and histopathologic features with GBM [12]. Tregs have been identified in high-grade canine oligodendrogliomas and astrocytoma [18, 19]. Moreover, we have demonstrated that CCL2 is robustly increased in canine HGG tumors relative to normal canine brain [14]. Importantly, the CCL2-CCR4 axis is targetable and efficacious in canine patients. Blockade of CCR4 with the FDA approved monoclonal antibody reduced tumoral Treg infiltration and improved survival time in dogs affected with bladder and prostate cancer [20–21]. Small molecule inhibitors of CCR4 are also in development (AZD2098 and K777) [22, 23].

While Tregs have been observed in spontaneously occurring canine HGG, the mechanisms leading to their recruitment and function remains a critical knowledge gap for translational therapies targeting Tregs. Here, we performed a series of in vitro experiments using acutely isolated canine lymphocytes from healthy dogs and canine patient-derived glioma cell lines to interrogate the CCL2-CCR4 axis in dogs. Canine Tregs increased migration toward human recombinant CCL2 and glioma cell line-derived supernatant. However, this effect was abolished in the presence of CCL2 and CCR4 blockade. Moreover, canine glioma cells increased ***CCL2*** mRNA expression when exposed to Tregs, but not CD4 + helper T-cells. These data demonstrate that the CCL2-CCR4 signaling axis is necessary and sufficient for canine glioma cell-induced Treg migration and is mechanistic contributor of Treg recruitment to the tumor microenvironment in canine HGG. This work provides mechanistic rationale for preclinical trials testing the efficacy of CCR4^+^Treg-targeted therapies in canine HGG.

## Materials & Methods

### Isolation of canine T regulatory cells.

This study was approved by Institutional Animal Care and Use Committee and the UC Davis Veterinary Medical Teaching Hospital Clinical Trial Review Board. Three apparently healthy client-owned dogs with a normal physical examination were recruited for this study. Following client consent, peripheral blood samples were taken from the jugular or lateral saphenous veins using standard veterinary technique and collected in ethylenediaminetetraacetic acid (EDTA) tubes. Peripheral blood mononuclear cells (PBMCs) were isolated from whole blood by gradient separation and negative selection with ammonium chloride potassium (ACK) as previously described [24]. Purified cells were washed twice in PBS with 10% FBS by centrifuging at 600 g for five minutes at 4°C. After washing, cells were re-suspended in PBS with 10% FBS and prepared for fluorescence-activated cell sorting (FACS). Freshly isolated PBMCs were stained with mixtures of Pacific Blue (PB) conjugated anti-dog CD4 (clone YKIX302.9; Thermo Fischer Scieintific) and Phycoerythrin (PE) conjugated anti-dog CD25 (clone P4A10; Thermo Fischer Scientific). Cells were washed twice with PBS at room temperature for 10 minutes prior to sorting with the Beckman Coulter “MoFlo Astrios EQ”. CD4 + cells were sorted by expression of CD25 into CD4 + CD25high and CD4 + CD25low populations. CD25 expression in the 90th percentile (highest 10%) were defined as CD4+CD25high, whereas CD25 expression at or below the 20th percentile (lowest 20%) were defined as CD4 + CD25low. Cells were immediately re-suspended in culture medium after sorting and seeded into 96-well plates at a concentration of 0.5–2 × 10^4^ cells per well. Cells were cultured in 5% reduced serum 1640 RPMI for 8–10 hours prior to downstream assays. Media contained RPMI-1640 complemented with 10% FBS, 10mM HEPES, 100μg/mL streptomycin, 100U/mL penicillin, 2000U/ml recombinant IL-2 and 0.5mM β-mercaptoethanol.

### Flow Cytometry.

Freshly isolated canine PBMCs underwent flow cytometry analysis using monoclonal antibodies (mAbs) against canine-specific or cross-reactive antigens. The following antibodies were utilized: CD3 (clone CA17.2A12; BioRad), CD4 (clone YKIX302.9; Thermo Fisher Scientific), CD8 (clone YCATE55.9, BioRad), CD25 (clone P4A10, Thermo Fisher Scientific), CCR4 (clone 1G1; BD Biosciences), and FOXP3 (clone FJK-16s; eBioscience). Viability of freshly isolated cells was performed with live/dead fixable Near-IR cell staining (# L34976, Thermo Fisher Scientific).

### FOXP3 visualization in CD4 + CD25 ^high^ T lymphocytes.

CD4^+^CD25high cells underwent FOXP3 intracellular staining with e-fluor 660 - conjugated anti-dog FOXP3 (clone FJK-16s; eBioscience). Intracellular staining, permeabilization, and fixation were performed using a commercially available transcription factor staining buffer set according to the manufacturer instructions (# 00-5523-00, eBioscience^™^). Cells were mounted with Vectashield with 4’,5-diamidino-2phenylindole (DAPI) (Vector Labs, Burlingame, CA, USA). Confocal imaging was performed on a Leica SP8 STED 3X microscope (objective 63x).

### Canine Glioma Cell Lines.

We utilized four patient derived canine high grade astrocytoma cell lines in this study (J3T-Bg, G06A, GSC 1110, and GSC 0514) [25, 26]. GSC 0514 and GSC 1110 lines are considered canine glioma stem cell like cells and have been verified to express neural progenitor cell markers (SOX2, OLIG2, GFAP, NES) [25]. Genomic integrity of each cell line was verified by comparison of copy number alterations with parental tumor DNA using Illumina Canine HD SNP array and sequenced to confirm canine origin. All cell lines were routinely tested and confirmed to be mycoplasma free by PCR. GSC1110 and GSC0514 cell lines were cultured in the serum free conditions containing Neurobasal-A media (Thermo Fisher Scientific; 10888022), B27 supplement (Thermo Fisher Scientific; 12587010), N2 supplement (Thermo Fisher Scientific; 17502048), and 0.5X L-glutamine (Thermo Fisher Scientific; 25030081). bFGF (R&D Systems, Inc., Minneapolis, MN, USA; 233-FB-025), EGF (R&D Systems, Inc., USA; 236-EG-200), and PDGF (Gemini Bio Products, West Sacramento, CA, USA; 300–178P) were added at 25 ng/ml concentration. J3T-Bg and G06A cell lines were cultured in media consisting of Dulbecco’s Modified Eagle Medium with high glucose and glutamine, supplemented with 10% heat-inactivated fetal calf serum and 100 units/mL of penicillin, 100 μg/mL of streptomycin.

### Transwell Migration Assays.

The modified Boyden chamber assay was used for analysis of Treg chemotaxis as previously described [9–11]. Tregs (3.1×10^4^), suspended in 5% reduced serum 1640 RPMI media, were seeded into a 24-well plate with 5μm pore polycarbonate filter inserts (Corning, Corning, NY). Bottom chambers contained 1) human recombinant CCL2 (rhCCL2; 0.5 ug/ml) in complete 1640 RPMI media, 2) J3TA, or GSC 0514 cell supernatant obtained at 80% confluency, or 3) complete 1640 RPMI media only. Additional experiments were repeated following a 30-minute incubation of media with 1) anti-CCL2 antibody (5.0 ug/ml; clone # 280702, R&D Systems) (rhCCL2 experiments only) or 2) pre-treatments of Tregs with CCR4 antagonist C 021 dihydrochloride (5 uM, Tocris). Following 6-hour incubation (37°C, 5% CO_2_), cells in the bottom chamber were manually counted. Dead cells were excluded by trypan blue staining. Two independent experiments with technical duplicates per condition were performed.

### Treg-Glioma cell Co-culture.

Canine T lymphocytes (3.1×10^4^; Tregs or CD4 + helper T cells), suspended in complete 1640 RPMI media, were seeded into a 24-well plate with 3μm pore polycarbonate filter inserts (Corning, Corning, NY) and exposed to canine glioma cells (GSC 1110, GSC 0514, J3T-Bg, or G06A) at 80% confluency in the bottom chamber. Following 24-hour incubation (37°C, 5% CO_2_), glioma cell lines were washed twice with PBS and RNA was isolated as previously described [11].

### Quantitative Real-Time PCR.

Total RNA was isolated from glioma cells using the Direct-zol MiniPrep kit (Zymo Research, Irvine, CA, USA) according to manufacturer’s instructions. DNase treatment was carried out on the column before RNA elution. Using one microgram purified RNA, cDNA was reverse transcribed using the high-capacity cDNA reverse transcription kit (Thermo Fisher – Applied Biosystems, Waltham, MA, USA). We have used previously validated in dogs sets of primers [15, 21, 27]. Primer pair sequences were as follows: CCL2: 5′-GAGATCTGTGCTGACCCCAAA-3′ (forward) and 5′-TTGCAGTTTGGGTTTGGCTTT – 3^1^ (reverse); CCR4: 5′-CCC TAA GCC TTGCAC CAA AGA-3′ (forward) and 5′-TGT ACT TGA ACA GGA CCA CAA CCA-3′ (reverse); FOXP3: 5′ - GTCTTCGAGGAGCCAGAGGA-3^1^ (forward) and 5′ - GCACCCAGCTTCTCCTTCTC-3′ (reverse); GAPDH: 5′ TGTCCCCACCCCCAATGTATC-3′ (forward) and 5’-CTCCGATGCCTGCTTCACTACCTT-3’ (reverse). qPCR reactions consisted of primer pairs at a final concentration of 200nM, 50ng cDNA template, and SSoAdvanced Universal SYBR Green Supermix (Bio-Rad, Hercules, CA, USA) per manufacturer’s protocol. Reactions were carried out on a CFX connect machine (Bio-Rad) with a three-step cycle of 95°C-15s, 60°C-20s, 72°C-20s, followed by a melt curve ramp from 65°C to 95°C. Data were acquired during the 72°C step, and every 0.5°C of the melt curve. All reactions were run as 20μl triplicates for glioma cell or 20μl duplicates for T-cells, and the average Cq used as the data point for a given sample. Care was taken at each step to minimize assay variability: samples were processed in parallel; the same batch of reverse transcriptase was used for all samples. mRNA expression values were quantified by the 2-ΔΔCt method, whereby ΔCT = 18S Ct – gene of interest Ct.

## Statistical analysis.

All statistical analyses were performed using Graphpad Prism 9 (GraphPad Software Inc., San Diego CA). Data were tested for normality via Shapiro-Wilks test. Data are reported as mean ± SEM. Statistical significance was assessed via unpaired two-tailed student’s t test or ANOVA with Tukey’s multiple comparisons test. Results were regarded statistically significant for p < 0.05.

## Results

### High CD25 expression correlates with robust FOXP3 expression in CD4 + positive canine T lymphocytes.

**Figure 1:**
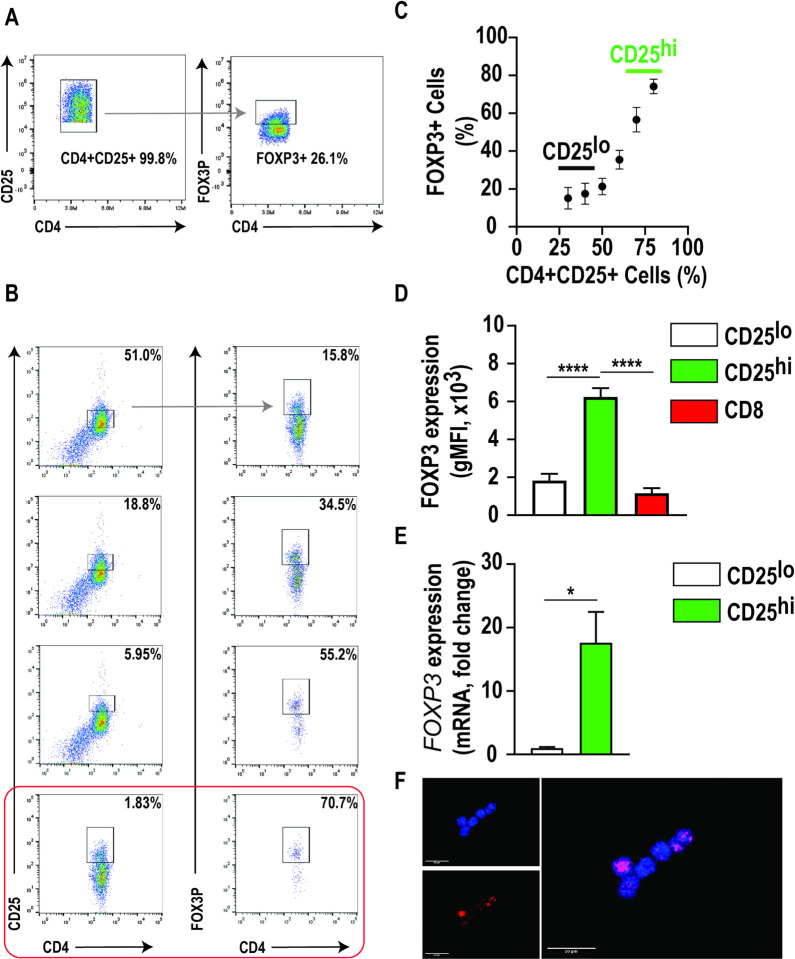
High CD25 expression correlates with robust FOXP3 expression in CD4+ positive canine T lymphocytes. **a)** Representative flow cytometric plot illustrating a low percentage (26.1%) of FOXP3+ cells when gating includes all CD25+CD4+ cells. **b)** Representative flow cytometric plot illustrating a positive correlation between FOXP3 expression and CD25 expression in CD4+ canine T lymphocytes. In this example, FOXP3 expression was identified in 70.7% of cells with the highest CD25 expression (1.83%; red frame). **c)** Scatter dot plot demonstrating the positive correlation between FOXP3 and CD25 expression. **d)** FOXP3 geometric mean fluorescence intensity and **e)** mRNA expression was significantly increased in CD4+ CD25high T lymphocytes compared to CD4+ CD25high and CD8+ T lymphocytes. **f)** Positive immunoreactivity for FOXP3 was observed co-localizing with nuclei of CD4+CD25high T lymphocytes. Three biological replicates were tested (n=3 dogs) with three technical replicates per sample. Comparisons based on one-way ANOVA with Tukey’s multiple comparisons test; bars represent group mean with standard error of the mean (SEM). ****p<0.0001.

There is very limited data on identification and isolation of Tregs for downstream assays in dogs [28]. We hypothesized that freshly isolated canine CD4^+^CD25^+^ T cells would possess the FOXP3 transcription factor associated with the T regulatory cell, similar to human Tregs. To test this hypothesis, we assessed FOXP3 expression in acutely isolated CD4^+^CD25^+^ T cells from healthy dogs. When the total population of CD4^+^CD25^+^ cells were gated together, FOXP3 immunoreactivity was detected in 26.1% (33.4 ± 5.7, n = 3) of cells (Fig. 1A). However, when examining the expression of FOXP3 as a function of CD25 expression, we observed a positive correlation between CD25 expression and FOXP3 expression (Fig. 1B, C). The geometric mean fluorescence intensity (gMFI) of FOXP3 in the CD4^+^CD25^high^ population was significantly increased compared to the CD4^+^CD25^low^ and CD8^+^ cellular populations (p < 0.0001) (Fig. 1D). Furthermore, we observed a 17-fold increase in *FOXP3* mRNA expression in CD4^+^CD25^high^ cells compared to CD4^+^CD25^low^ cells (p < 0.05) (Fig. 1E). Immunocytochemistry confirmed intranuclear FOXP3 immunoreactivity in CD4^+^CD25^high^ T cell population (Fig. 1F). Therefore, these data demonstrate that CD4^+^CD25^high^ T cells are indeed FOXP3^+^ expressing cells and positive selection of this population is a viable approach to isolate canine Tregs in the absence of intranuclear transcription factor FOXP3 immunostaining.

### CD4 + CD25high lymphocytes are enriched with CCR4.

**Figure 2:**
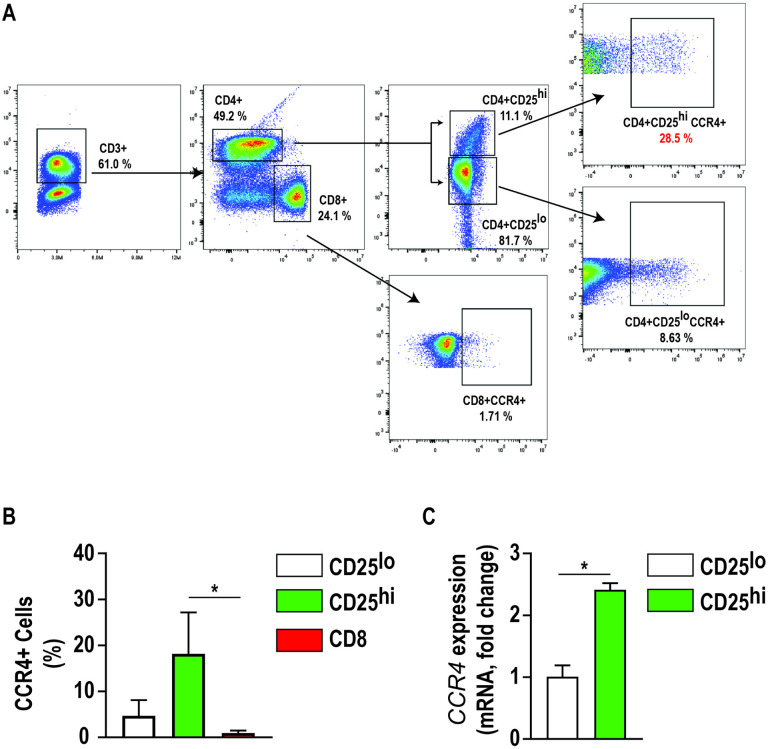
Canine Tregs has the highest expression of CCR4 amongst T-cells. **a)** Representative flow cytometric plot illustrating percentage of CCR4+ cells within CD4+CD25+ and CD8+ canine T lymphocytes. In this example, CCR4 expression was identified in 28.5% of CD4+ CD25high cells and 8.6% of CD4+ CD25low cells. The expression of CCR4 in CD8+ T lymphocytes was minimal, 1.71%. **b)** The percentage of CCR4+ cells identified via flow cytometry and **c)** mRNA expression was significantly increased in CD4+CD25high T lymphocytes, (2.43-fold increase) compared to CD4+CD25low and CD8+ T lymphocytes. Three biological replicates were tested (n=3 dogs) with three technical replicates per sample. Comparisons based on one-way ANOVA with Tukey’s multiple comparisons test; and unpaired two-tailed student’s t test, bars represent group mean with standard error of the mean (SEM). *p<0.05.

Cellular CD25 expression was associated with enrichment of CCR4 expression on freshly isolated canine CD4 + lymphocytes (Fig. 2A). The proportion of cells expressing CCR4 was significantly increased in CD4^+^CD25^high^ population compared to the population of CD8^+^ cells (p < 0.05, n = 3) (Fig. 2B). *CCR4* mRNA was 2.4-fold increased in CD4^+^CD25^high^ cells compared to CD4^+^CD25^low^ cells (p < 0.05) (Fig. 2C). Thus, we positively selected CD4^+^CD25^high^ lymphocytes, which express robust FOXP3 and are enriched for CCR4, to identify and isolate putative Treg cells for downstream assays.

### The CCL2-CCR4 axis induces chemotaxis in canine Tregs.

**Figure 3:**
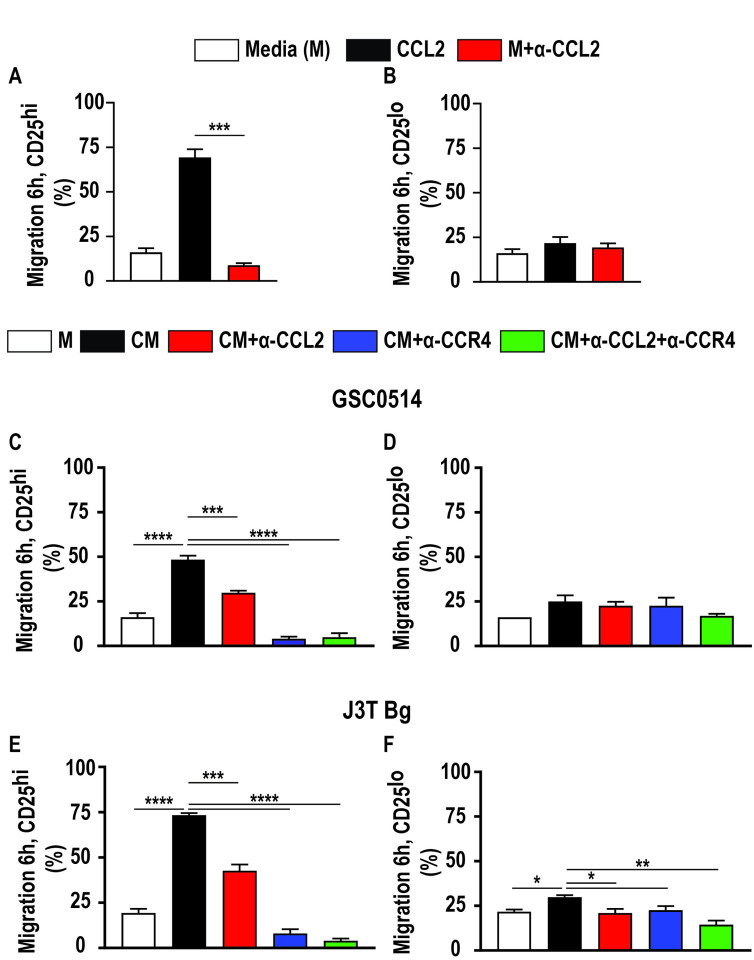
The CCL2-CCR4 axis induces chemotaxis in canine Tregs. **a)** Acutely isolated canine Tregs demonstrated robust chemotaxis toward human recombinant CCL2 (rhCCL2; 0.5 ug/ml), relative to media alone (p=0.0007). This effect was abolished with the addition of anti-CCL2 antibody (5.0 ug/ml; p=0.15). **b)** Migration of conventional CD4+ T cells was not affected by rhCCL2 (p=0.2) or anti-CCL2 antibody (p=0.4). c) Chemotaxis of acutely isolated canine Tregs (CD4+CD25high) increased toward GSC0514 derived supernatant (p<0.0001) and e) J3T Bg derived supernatant (p<0.0001). This effect was mitigated with the addition of anti CCL2 antibody (GSC0514, p=0.006; J3T Bg, p=0.0001). This effect was abolished following Treg pre-treatment with CCR4 inhibitor (GSC0514 p<0.0001; J3T Bg p<0.0001) and a combination of anti-CCL2 and CCR4 inhibition (GSC0514 p<0.0001; J3T Bg p<0.0001). Migration of conventional CD4+ T cells was not affected by d) GSC0514 derived supernatant (p=0.06) nor perturbation of CCL2 (p=0.8), CCR4 (p=0.8) vs combination of the two signaling (p=0.09). Chemotaxis of conventional CD4+ T cell towards J3T Bg supernatant was induced, f) (p=0.02) and this effect was mitigated by anti-CCL2 antibody f) p=0.016; CCR4 inhibitor f) p=0.03 and combination of both f) p=0.001). Comparisons based on one-way ANOVA with Tukey’s multiple comparisons test; bars represent group mean with standard error of the mean (SEM). ****p<0.0001.

Freshly isolated canine Tregs (CD4^+^CD25^high^) were utilized to determine whether CCL2 is necessary and sufficient to induce canine Treg chemotaxis. Indeed, acutely isolated canine Tregs demonstrated robust chemotaxis toward human recombinant CCL2 (rhCCL2), with a 53.2% increase in migration relative to media alone (Fig. 3A; p = 0.0007). This effect was abolished with the addition of anti-CCL2 antibody (5.0 ug/ml) (Fig. 3B). Similarly, supernatant from GSC0514 (Fig. 3C; p < 0.0001) and J3T Bg (Fig. 3E; p < 0.0001) canine glioma cell lines induced a robust increase in Treg chemotaxis (32.2% and 54.03%, respectively), which was mitigated by addition of anti-CCL2 antibody (Fig. 3C, GSC0514 p = 0.006; Fig. 3E, J3T Bg p = 0.0001). Strikingly, Treg chemotaxis toward glioma cell supernatant was abolished following pre-treatment with CCR4 inhibitor (Fig. 3C, GSC0514 p < 0.0001; Fig. 3E, J3T Bg p< 0.0001), as well as combination treatment with an anti-CCL2 antibody and CCR4 inhibitor (Fig. 3C, GSC0514 p < 0.0001; Fig. 3E, J3T Bg p< 0.0001). CD4 + helper T cell chemotaxis was not affected by rhCCL2, GSC cell supernatant, nor perturbation of CCL2 or CCR4 signaling (Fig. 3D, p = 0.09). Chemotaxis of CD4 + helper T cell towards J3T Bg supernatant was minimally induced (8.06% increase) (Fig. 3F, p = 0.02) and this effect was mitigated by anti-CCL2 antibody (Fig. 3F, p = 0.01), CCR4 inhibitor (Fig. 3F, p = 0.03), and combination of both (Fig. 3F, p = 0.001). Taken together, these data support that the CCL2-CCR4 signaling axis is necessary and sufficient for canine Treg chemotaxis.

### Canine Tregs induces increased CCL2 mRNA expression in canine glioma cell lines.

**Figure 4:**
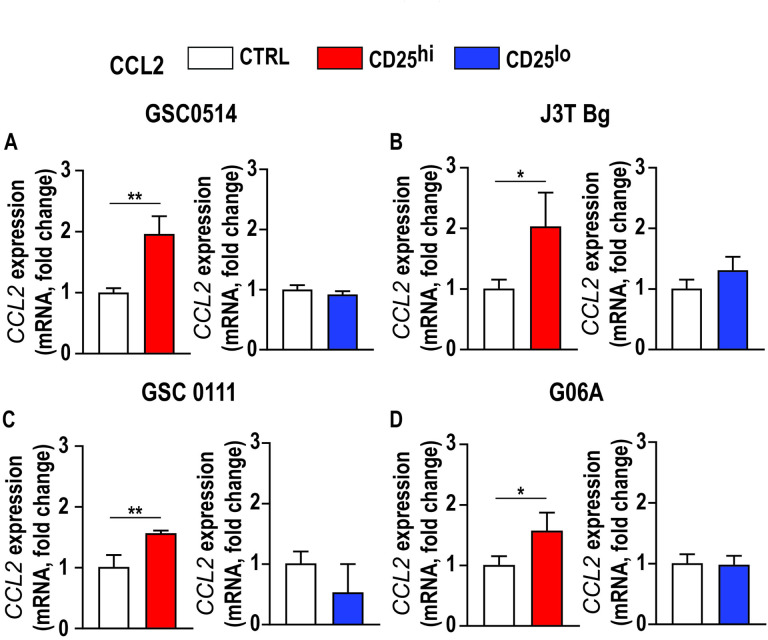
Canine glioma CCL2 mRNA expression increases when exposed to Tregs but not conventional T-cells. mRNA levels of *CCL2* were increased in **a)** GSC0514, 0.95 ± 0.17-fold increased, p<0.01; **b)**J3T Bg, 1.02 ± 0.33-fold increased, p<0.05; **c)** GSC0111, 0.55 ± 0.11-fold increase, p<0.01; **d)** G06A, 0.56 ± 0.19-fold increase, p<0.05, co-cultured with CD4+CD25high T lymphocytes relative to GSC0514, J3T Bg, GSC0111 and G06A cell lines without exposure to CD4+CD25high. mRNA levels of ***CCL2*** were not significantly different when glioma cells were co-cultured with conventional T-cells. **a)** 0.08 ± 0.05-fold decrease in GSC0514, **b)** 0.30 ± 0.15 - fold increase in J3T-Bg, **c)** 0.47 ± 0.29-fold decrease in GSC1110 and d) 0.02 ± 0.1-fold decrease in G06A cell line. Comparisons based on unpaired student’s t test; bars represent group mean with standard error of the mean (SEM).

Glioma cells increase secretion of CCL2 to recruit immune-regulatory cells in models of human glioma [27]. To determine if canine Tregs are capable of stimulating CCL2 expression in canine glioma cells, we performed co-culture experiments. Following 24-hour co-culture with Tregs, we observed increased *CCL2* mRNA expression in each of four canine glioma cell lines (GSC0514: 1.02± 0.33, Fig. 4A p = 0.005; J3T-Bg: 1.02 ± 0.33, Fig. 4B p = 0.03; GSC1110: 0.55 ±0.11, Fig. 4C p = 0.008; and G06A: 1.02 ± 0.33, Fig. 4D p = 0.04). *CCL2* mRNA expression was not altered in any glioma cell line following co-culture with CD4 + helper T cells (Fig. 4A, B, C, D).

## Discussion

Spontaneously occurring glioma in pet dogs is increasingly recognized as a unique model with valuable translational potential [29–31]. Canine preclinical research and clinical trials may allow fast-track clinical investigations in human patients [29, 32]. Here, we have demonstrated that CCL2-CCR4 is a relevant, targetable, bi-directional Treg-glioma signaling axis in the dog. Our studies confirmed that canine glioma cells induced Treg chemotaxis through CCL2-CCR4 signaling, which was abolished following blockade. Given the marked immunosuppression induced by Tregs in the glioma microenvironment, targeting CCL2-CCR4 can potentially improve patient outcomes through synergy with existing conventional and other experimental glioma therapies. As CCR4 monoclonal antibodies are FDA-approved for the treatment of Sezary syndrome, an aggressive human cutaneous T cell lymphoma [28], clinical investigations in canine glioma patients have the potential to support studies in human glioblastoma patients.

Since the discovery of Tregs and their immunosuppressive, pro-cancerous properties, considerable effort has gone into defining, characterizing, and targeting murine and human Treg cells [31]. In recent years, the successful reversal of Treg-mediated immunosuppression has advanced approaches to human oncology [34–36]. Depleting or inhibiting intratumoral Treg influx in murine models of adenocarcinoma, lymphoma, colon carcinoma, melanoma, and thymoma reduced tumor growth and, in some cases, induced remission [34–39]. Phase 1 and II clinical trials investigating Treg-targeted therapies are currently underway in several cancer types [NCT03236129, NCT00986518, NCT05200559, NCT00847106, NCT02009384]. The work of several groups has shown that these immunosuppressive cells support gliomagenesis [38]. Blockade of Treg mobilization increases the survival of glioma-bearing mice, showing antineoplastic potential of targeting the recruitment capacity of these cells in glioblastoma [10, 11, 38, 39]. Canine Treg characterization is extremely limited [40]. Given the scarcity of information on the identification and isolation of canine Tregs, one of the objectives of our work was to establish selection criteria for the isolation of live dog Tregs to develop preclinical support for clinical studies in dogs and subsequently in human patients.

There is considerable overlap in surface marker expression between Tregs and helper T cells. Moreover, while overexpression and nuclear localization of the forkhead box transcription factor three (FOXP3) by CD4 + CD25 + T cells demonstrate high specificity for Tregs, detection requires cellular permeabilization and is not suitable for isolating live Tregs. Similarly, as in human Tregs, we observed that the total population of CD4^+^CD25^+^ canine T cells harvested from freshly isolated PBMCs was poorly enriched in FOXP3 (Fig. 1A). However, cascaded gating via flow cytometry revealed a positive correlation between CD25 and FOXP3. Over 70% of these cells with the highest CD25 expression (90th percentile) also expressed FOXP3 (Fig. 1B). Therefore, high CD25 expression is a critical marker for identifying canine Tregs and can serve as a surrogate marker for isolating these cells. This gating strategy also proved to be a robust workflow for the selective isolation of canine CD4 + CD25 + expressing high levels of CCR4 and using the enriched Tregs for downstream assays from freshly isolated canine PBMCs.

Our group and others have previously shown that glioma derived CCL2 is one of the crucial chemoattractant responsible for Treg recruitment into the glioma microenvironment in murine models and human tissues [10, 11, 16]. An increased CCL2 expression has been correlated with reduced overall survival in human glioma patients [11]. Importantly, antibody-mediated targeting of the Treg CCL2 high-affinity receptor, CCR4, reduced intratumoral Treg abundance and prolonged survival in a mouse glioma model [10, 11]. Our work also demonstrated that CCL2 is robustly increased in high-grade canine astrocytoma compared to normal brains and given the role of CCL2 and CCR4 in both human and mouse models of gliomas, we studied here how this increased expression of CCL2 may affect the canine Tregs and CD4^+^ helper T-cells.

We performed a series of migration experiments to interrogate the CCL2-CCR4 axis in dogs. Importantly, canine Tregs migration was significantly enhanced by canine glioma cell line-derived supernatant, which was mitigated by the presence of an anti-CCL2 antibody. However, the most significant reduction in canine Treg migration was observed in the presence of a CCR4 antagonist or when combined with an anti-CCL2 antibody (dual-blockade). This suggests that the CCR4 receptor may be the major molecule mediating Treg migration to the tumor site in response to CCL2, and possibly other CCR4 ligands [41] secreted in the tumor environment. In the veterinary literature, several other chemokines are capable of binding and inducing Treg migration via the CCR4 receptor. For instance, CCL17 was reported to bind the CCR4 receptor to trigger the migration of Tregs toward canine urothelial carcinoma [42]. These chemokines have not been the subject of our study, and we focus on the interrogation of the CCL2-CCR4 axis in canine gliomas as the next translational, targetable step of our work. A comprehensive evaluation of Treg-responsive chemokines within canine glioma supernatant and tumor microenvironment will be an important next-step to advance understanding of Treg biology in the context of high-grade glioma.

Here, we have interrogated the CCL2-CCR4 axis in canine glioma. Our data indicate that targeting either CCL2 or CCR4, alone or in combination, may represent a viable therapeutic strategy to diminish Treg accumulation in the glioma microenvironment. The availability of several therapeutic and experimental agents, including FDA-approved anti-CCR4 monoclonal antibody (Mogamulizumab) and small molecule inhibitors such as FLX475 [28, 43] AZD2098 and K777 [21, 22, 23, 43], facilitates preclinical trials in canines to determine safety, efficacy, and whether this approach may have synergy with other immunotherapies. Given the importance of host immune mechanisms in governing the response to immunotherapy, future canine clinical trials will be critical to inform therapeutic efficacy in human patients.

In conclusion, we have established a workflow to identify and positively select canine Tregs from whole blood in dogs for downstream use. We have further established that the CCL2-CCR4 signaling axis is necessary and sufficient for canine Treg chemotaxis. Importantly, this work demonstrates that canine HGG is a viable preclinical model to explore immunotherapeutic approaches targeting this axis. Efficacy in canine patients will pave the way for promising clinical trials for human patients.

## Figures and Tables

**Figure 5:**
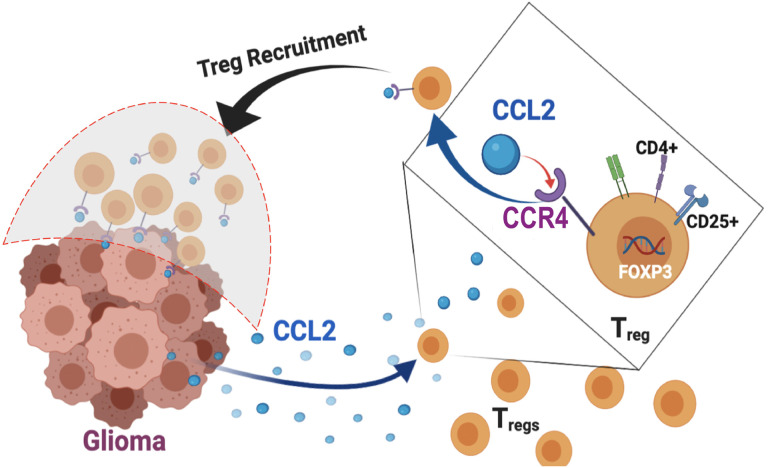
Schematic diagram illustrating validated hypothesis. CCL2-CCR4 axis as a bi-directional Treg-glioma immunosuppressive and tumor-promoting axis in canine high-grade glioma.
